# HIV-1 Replication Fitness of HLA-B*57/58:01 CTL Escape Variants Is Restored by the Accumulation of Compensatory Mutations in Gag

**DOI:** 10.1371/journal.pone.0081235

**Published:** 2013-12-05

**Authors:** Esther F. Gijsbers, K. Anton Feenstra, Ad C. van Nuenen, Marjon Navis, Jaap Heringa, Hanneke Schuitemaker, Neeltje A. Kootstra

**Affiliations:** 1 Department of Experimental Immunology, Sanquin Research, Landsteiner Laboratory, and Center for Infectious Diseases and Immunity Amsterdam (CINIMA), Academic Medical Center, University of Amsterdam, Amsterdam, The Netherlands; 2 Centre for Integrative Bioinformatics (IBIVU) and Amsterdam Institute for Molecules, Medicines and Systems (AIMMS), VU University, Amsterdam, The Netherlands; 3 Netherlands Bioinformatics Centre (NBIC), Nijmegen, The Netherlands; INSERM, France

## Abstract

Expression of HLA-B*57 and the closely related HLA-B*58:01 are associated with prolonged survival after HIV-1 infection. However, large differences in disease course are observed among HLA-B*57/58:01 patients. Escape mutations in CTL epitopes restricted by these HLA alleles come at a fitness cost and particularly the T242N mutation in the TW10 CTL epitope in Gag has been demonstrated to decrease the viral replication capacity. Additional mutations within or flanking this CTL epitope can partially restore replication fitness of CTL escape variants. Five HLA-B*57/58:01 progressors and 5 HLA-B*57/58:01 long-term nonprogressors (LTNPs) were followed longitudinally and we studied which compensatory mutations were involved in the restoration of the viral fitness of variants that escaped from HLA-B*57/58:01-restricted CTL pressure. The Sequence Harmony algorithm was used to detect homology in amino acid composition by comparing longitudinal Gag sequences obtained from HIV-1 patients positive and negative for HLA-B*57/58:01 and from HLA-B*57/58:01 progressors and LTNPs. Although virus isolates from HLA-B*57/58:01 individuals contained multiple CTL escape mutations, these escape mutations were not associated with disease progression. In sequences from HLA-B*57/58:01 progressors, 5 additional mutations in Gag were observed: S126N, L215T, H219Q, M228I and N252H. The combination of these mutations restored the replication fitness of CTL escape HIV-1 variants. Furthermore, we observed a positive correlation between the number of escape and compensatory mutations in Gag and the replication fitness of biological HIV-1 variants isolated from HLA-B*57/58:01 patients, suggesting that the replication fitness of HLA-B*57/58:01 escape variants is restored by accumulation of compensatory mutations.

## Introduction

HLA class I alleles are associated with the clinical course of HIV-1 infection. HLA-B*57 and the closely related HLA-B*58:01 are overrepresented in so called long-term nonprogressors (LTNPs) [1]–[7]. Individuals carrying these HLA alleles exert strong cytotoxic T lymphocyte (CTL) responses against conserved viral regions such as Gag, resulting in immunological control of HIV-1 replication and a slower disease progression [8]–[13]. However, HIV-1 continuously adapts to the high selective pressure exerted by the immune system, resulting in escape mutations in viral epitopes and loss of recognition by CTLs [14]–[22]. Although the escape mutations allow evasion of the CTL response, viral escape is not always associated with disease progression. Some CTL escape mutations come at a fitness cost, particularly when situated in conserved viral regions, and patients may benefit from a reduced viral replication capacity despite escape from CTL-mediated killing. The T242N escape mutation in the HLA-B*57/58:01 restricted TW10 epitope in Gag impairs viral replication, which can explain the protective effect of this HLA type even after escape from CTL responses has occurred [22]–[27]. Additional mutations within or flanking the TW10 epitope can have a compensatory effect and partially restore the fitness cost associated with the T242N mutation [25]–[28]. Although HLA-B*57 and HLA-B*58:01 are overrepresented in LTNPs, the majority of patients carrying these protective HLA alleles do show a progressive disease course in the absence of antiretroviral treatment.

Previously, we studied 5 HLA-B*57/58:01 LTNPs and 5 HLA-B*57/58:01 progressors longitudinally, and observed similar frequencies of HIV-1 Gag-specific CTL responses and dynamics in escape mutations in HLA-B*57/58:01-restricted CTL epitopes [25]. This indicates that Gag specific CTL responses and the prevalence of CTL escape mutations does not relate to the differential disease course in these patients. However, an increase in replication kinetics of viral variants isolated from progressors was observed during longitudinal follow-up [25]. Furthermore, an association between disease progression and the presence of 2 or more of the compensatory mutations H219Q, I223V, M228I, N252H and G248X was observed [25].

Here, we studied which compensatory mutations were involved in the restoration of the viral fitness of variants that escaped from HLA-B*57/58:01-restricted CTL pressure. Virus isolates from HLA-B*57/58:01 individuals contained multiple CTL escape mutations, and these escape mutations were not associated with disease progression. In the HLA-B*57/58:01 progressors, 5 additional mutations were observed in the Gag protein that increased the replication rate of the HLA-B*57/58:01 CTL escape variants.

## Results

### Sequence variation in Gag associated with HLA-B*57/58:01

To study differences in the Gag protein of virus isolates obtained from HLA-B*57/58:01 progressors and LTNPs that may explain the differences in replication kinetics, we compared Gag sequences (positions 1-364) obtained from various time points during the course of infection (Table 1 and Table S1) using the Sequence Harmony (SH) algorithm [29]–[31]. SH detects positions within an alignment that show differences in amino acid composition between two groups of sequences and analyses the frequency distribution of amino acid variation per position [29]–[31]. Low SH-scores indicate position where the amino acid compositions are different between the two groups; a score of 0 indicates that the amino acids at a given position are completely different between both groups, while the maximum score of 1 indicates that the amino acid compositions are indistinguishable.

**Table 1:** Characteristics of patients positive for HLA-B*57 or HLA-B*58:01 and the Gag sequences analyzed.

							Sequences	
Patient	Seroconversion (S) or HIV+ Entry (E) date	HLA A alleles		HLA B alleles		Δ32 CCR5 genotype	Time after seroconversion or study entry (months)	Number of sequences
L5	06-11-1984 (E)	A*26	A*68:01	B*57	B*07:02	Wild type	89	6
							177	6
L6	09-02-1988 (E)	A3	A11	B*58:01	B7	Heterozygous	17	5
							114	15
L7	03-12-1984 (E)	A*01	A*02	B*57:01	B*050:1	Heterozygous	26	5
							78	6
							102	8
							136	12
L8	06-02-1990 (S)	A*02	A*02	B*57:01	B*37:01	Wild type	70	2
							91	9
							137	6
L9	04-10-1985 (S)	A*01	A*68:02	B*57:01	B*14:02	Wild type	42	1
							59	1
							77	6
P9	20-01-1985 (E)	A2	A3	B*57:01	B*07:02	Wild type	9	8
							78	3
P10	01-01-1984 (E)	A*02	A*26	B*57:01	B*41:02	Wild type	69	7
							84	7
							123	9
P11	15-05-1990 (S)	A1	A11	B*57:01	B*08:01	Wild type	3	2
							25	9
							32	23
							69	13
P12	24-08-1992 (E)	A*01	A*26	B*57:01	B*38:01	Wild type	0.4	5
							48	11
							74	12
P13	22-01-1985 (E)	A*01	A*01	B*57:01	B*08:01	Wild type	3	6
							46	10

L: long term non-progressor; P: progressor. S: date of seroconversion for HIV-1 antibodies during active follow-up in the cohort. E: HIV+ entry into the cohort.

**Figure 1:**
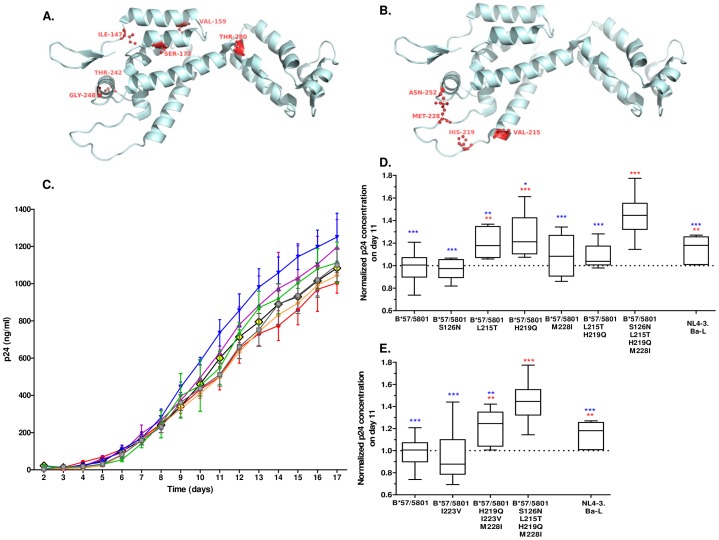
Sequence variation in Gag affects viral replication fitness. A. Sequence Harmony analysis detecting amino acid changes associated with the presence of HLA-B*57/58:01, irrespective of disease course. Longitudinal Gag sequences obtained from HLA-B*57/58:01 progressors and LTNPs (n = 10) were compared to Gag sequences obtained from patients negative for HLA-B*57/58:01 (n = 19). An SH value of 0.90 was used as cut-off. Six amino acid changes were identified to be specifically associated with the presence of HLA-B*57/58:01: I147L, V159I, S173T, T242N, G248A and T280V. Amino acid positions in capsid associated with HLA-B*57/58:01 are shown in red. B. Sequence Harmony analysis detecting amino acid changes associated with disease progression in HLA-B*57/58:01 patients. Gag sequences obtained from late in infection of HLA-B*57/58:01 progressors (n = 5) and HLA-B*57/58:01 LTNPs (n = 5) were compared. An SH value of 0.90 was used as cut-off. Five amino acid changes were identified to be associated with HLA-B*57/58:01 disease progression: S126N, L215T, H219Q, M228I and N252H. Amino acid positions in capsid associated with HLA-B*57/58:01 disease progression are shown in red. C. Replication kinetics (days 2–17) of constructed NL4-3.Ba-L viral variants containing mutations associated with HLA-B*57/58:01 (I147L, V159I, S173T, T242N, G248A and T280V; red), in combination with mutation S126N (orange), L215T (yellow), H219Q (purple), M228I (green), L215T and H219Q (grey), or a combination of all 5 compensatory mutations (S126N, L215T, H219Q, M228I and N252H; blue). Mean p24 concentrations (ng/ml) are given. Error bars represent the standard error of the mean. Data from one representative experiment are shown. D. Replication kinetics of NL4-3.Ba-L and constructed NL4-3.Ba-L viral variants containing mutations associated with HLA-B*57/58:01 (I147L, V159I, S173T, T242N, G248A and T280V) in the absence or presence of compensatory mutations. Normalized mean p24 values on day 11 were compared using the unpaired Student's T test. Statistical significance compared to the mutant virus carrying the mutations associated with the presence of HLA-B*57/58:01 are denoted in red, and significance compared to the virus carrying all mutations associated with the presence of HLA-B*57/58:01 and with disease progression is shown in blue. Statistical significance is indicated as follows: * p<0.05, ** p<0.01, *** p<0.0001. Error bars represent 2.5 – 97.5 percentiles. Data from one representative experiment are shown. E. Replication kinetics of NL4-3.Ba-L and constructed NL4-3.Ba-L viral variants containing mutations associated with HLA-B*57/58:01 (I147L, V159I, S173T, T242N, G248A and T280V) in the absence or presence of compensatory mutations described by Brockman *et al*. Normalized mean p24 values on day 11 were compared using the unpaired Student's T test. Statistical significance compared to the mutant virus carrying the mutations associated with the presence of HLA-B*57/58:01 are denoted in red, and significance compared to the virus carrying all mutations associated with the presence of HLA-B*57/58:01 and with disease progression is shown in blue. Statistical significance is indicated as follows: * p<0.05, ** p<0.01, *** p<0.0001. Error bars represent 2.5–97.5 percentiles. Data from one representative experiment are shown.

First, we identified amino acid variation in Gag specific for patients carrying HLA-B*57 or HLA-B*58:01 irrespective of their disease course. Therefore, we compared longitudinally obtained sequences from HLA-B*57/58:01 patients (n = 10: 5 progressors and 5 LTNPs, 221 sequences in total; Table 1 and Table S1) to longitudinally obtained sequences from patients negative for HLA-B*57 and HLA-B*58:01 alleles (n = 19, 152 sequences) using the SH algorithm with a cut-off value of 0.90. In this way, amino acid positions were considered to be different between the two groups when there was more than an approximate 10% difference in amino acid distribution at this position. In total, 6 amino acid positions in Gag were identified that showed significant variation in the dominant amino acid between sequences from individuals with or without HLA-B*57/58:01. Among these HLA-B*57/58:01 specific amino acid mutations were the HLA-B*57/58:01 associated CTL escape mutations T242N and G248A located in the TW10 epitope and the I147L mutation in the IW9 epitope. Additionally, the S173T adjacent to epitope KF11, and mutations V159I and T280V were associated with the presence of HLA-B*57/58:01 (Figure 1A, Table 2 and Table S1).

**Table 2:** Amino acid variation in Gag p17 and p24 specific for HLA-B*57/58:01.

AA	Comparison		AA variation***			
Position	SH		HLA-B*57/58:01		Non-B*57/58:01	
	SH value*	Z score**	Dominant	Minor	Dominant	Minor
I147	0.44	−120	L	MI	I	L
V159	0.80	−54	IV		V	I
S173	0.68	−65	ST		S	AT
T242	0.05	−400	N	TS	T	
G248	0.78	−47	GA	T	G	A
T280	0.88	−19	TV	SIA	T	VSA

*The Sequence Harmony (SH) score represents overlap in amino acid composition between the two groups analyzed (HLA-B*57/58:01 versus non-HLA-B*57/58:01). An SH value of 0 indicates no overlap (largest possible differences) while a value of 1 indicates complete overlap (no differences).

**All Z scores represent P values <0.0001 with correction for multiple testing (450 alignment positions).

***The most frequent amino acids found at a certain position are depicted as “Dominant”, whereas the “Minor” amino acids are present at least 2 times less frequent than the most frequently occurring amino acid at this position. Amino acids are ordered from highest to lowest frequency.

To identify sequence variation within Gag associated with disease progression, we compared sequences from viral variants obtained late in infection from 5 HLA-B*57/58:01 progressors (43 sequences; Table 1 and Table S1) and 5 HLA-B*57/58:01 LTNPs (45 sequences; Table 1 and Table S1) with SH, using a cut-off value of 0.90. Frequent variation in amino acid composition was observed at 5 positions within Gag (S126N, L215T, H219Q, M228I and N252H) in viral sequences obtained from progressors, whereas these amino acids were absent or only present at low frequency in sequences obtained from LTNPs (Table 3, Figure 1B and Table S1). When we repeated the SH analysis correcting for the number of sequences per patient to give each patient equal weight, the same amino acid residues showed significant variation between sequences obtained from LTNPs and progressors (data not shown).

**Table 3:** Amino acid variation in Gag p17 and p24 associated with HLA-B*57/58:01 disease progression.

AA	Comparison		AA variation***			
Position	SH		Progressors		LTNPs	
	SH value*	Z score**	Dominant	Minor	Dominant	Minor
S126	0.83	−10.7	SK	N	S	N
L215	0.61	−27.8	LT	I	L	M

*The Sequence Harmony (SH) score represents overlap in amino acid composition between the two groups analyzed (HLA-B*57/58:01 progressors versus HLA-B*57/58:01 LTNPs). An SH value of 0 indicates no overlap (largest possible differences) while a value of 1 indicates complete overlap (no differences).

**All Z scores represent P values <0.0001 with correction for multiple testing (450 alignment positions).

***The most frequent amino acids found at a certain position are depicted as “Dominant”, whereas the “Minor” amino acids are present at least 2 times less frequent than the most frequently occurring amino acid at this position. Amino acids are ordered from highest to lowest frequency.

### Mutations associated with disease progression in HLA-B*57/58:01 patients increase the viral replication kinetics *in vitro*

We hypothesized that the amino acid variation observed within Gag of viral variants obtained from HLA-B*57/58:01 progressors are compensatory mutations that increase the replication capacity of viral variants containing the CTL escape mutations. In order to study the effects of the sequence variation found, the mutations associated with disease progression (alone or in combination) were placed in the NL4-3.Ba-L molecular clone together with the 6 mutations specifically associated with the presence of HLA-B*57/58:01 (T242N, G248A, I147L, S173T, V159I and T280V) (Tables 2 and 3). The NL4-3.Ba-L molecular clone backbone already contains N252H, and this amino acid was not changed in the constructed viruses. The replication kinetics of the obtained viruses were analyzed on PHA stimulated PBMCs for 17 days (Figure 1C). An increase in viral replication was observed after introduction of the L215T and H219Q mutation (p = 0.002 and p = 0.0002 respectively, Figure 1D; Figure S1A), whereas mutations S126N and M228I did not alter replication kinetics (Figure1D and Figure S1A). The highest replication capacity was observed when the combination of all compensatory mutations was introduced in NL4-3.Ba-L carrying the HLA-B*57/58:01-associated mutations (p<0.0001, Figure 1D; Figure S1A). As mutations L215T and H219Q show the highest increase in replication capacity analyzing the single mutations, we also introduced a combination of these two mutations in the mutant virus carrying the 6 HLA-B*57/58:01-specific mutations. The combination of these two compensatory mutations, however, did not significantly increase viral replication kinetics compared to that HLA-B*57/58:01 background virus (Figure 1D and Figure S1A).

A previous study by Brockman *et al.* reported that the compensatory mutations H219Q, I223V, M228I, G248A and N252H partially restored replication kinetics of NL4-3 virus carrying the T242N escape mutation [27]. Three of these mutations (H219Q, M228I, N252H) were also associated with disease progression in our analysis, while the G248A mutation was found in all HLA-B*57/58:01 patients irrespective of their disease course. The I223V mutation was not observed to be different between LTNPs and progressors in our analysis, nor was it associated with the presence of HLA-B*57/58:01. To compare the effect of the compensatory mutations described by Brockman *et al.* and our present analysis on the replication fitness of the virus, we placed the described mutations in the NL4-3.Ba-L carrying the HLA-B*57/58:01-specific mutations (Table 2). The I223V mutation alone had no significant effect on the replication kinetics of NL4-3.Ba-L carrying the HLA-B*57/58:01 specific mutations (Figure 1E and Figure S1B). The combination of mutations described by Brockman *et al.* did indeed increase the replication kinetics of the NL4-3.Ba-L carrying the 6 HLA-B*57/58:01-specific mutations (p = 0.0012, Figure 1E; Figure S1B). However, the replication capacity of this virus was significantly lower than that observed for the virus carrying all of the mutations associated with HLA-B*57/58:01 disease progression identified in our present study (p = 0.0082, Figure 1E; Figure S1B). These findings suggest an additive effect of additional compensatory mutations on viral replication.

### The total number of escape and compensatory mutations in Gag correlates with the replication kinetics of biological HIV-1 isolates obtained from HLA-B*57/58:01 patients

**Figure 2:**
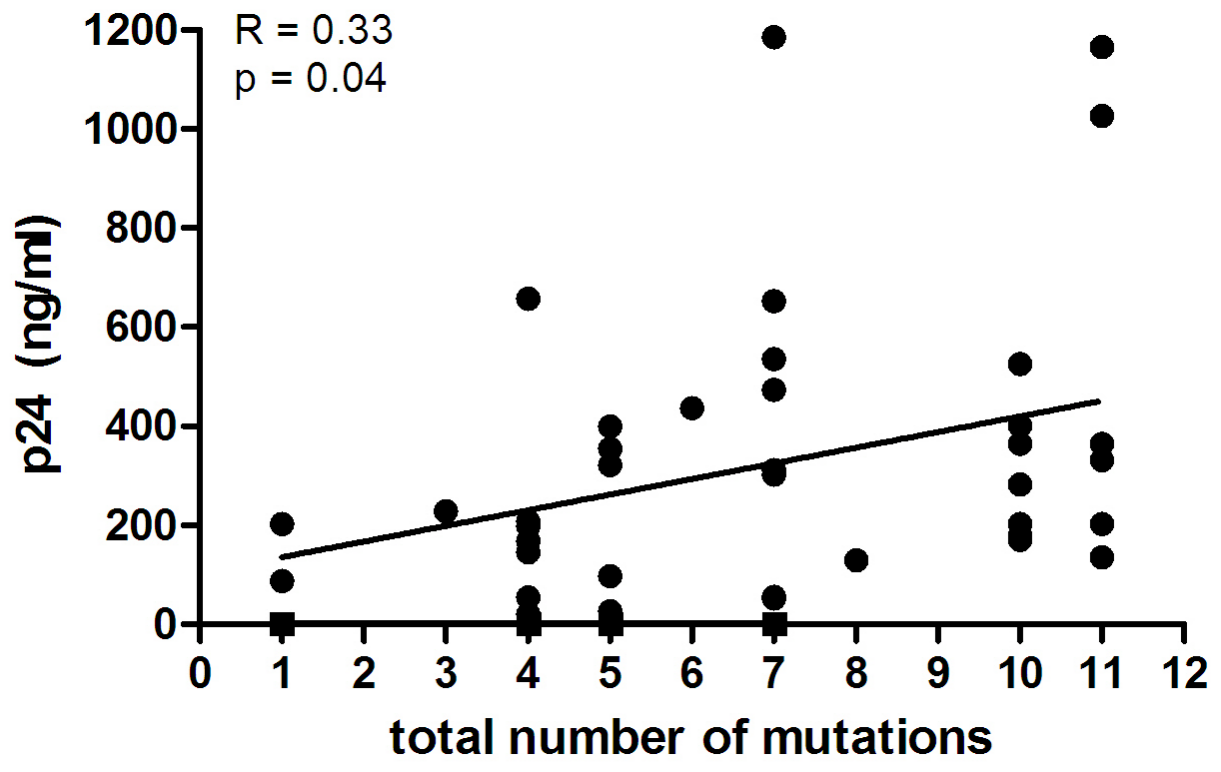
Accumulation of mutations in Gag correlates with a higher replication fitness. Spearman correlation analysis of the total number of escape and compensatory mutations associated with HLA-B*57/58:01 and the maximum p24 value observed for biological HIV-1 variants obtained from HLA-B*57/58:01 progressors and LTNPs in an *in vitro* replication assay.

During the course of infection, mutations accumulate in the virus as a result of the continuous selection for the fittest variant. Our results may suggest that compensatory mutations restore viral fitness in a cumulative manner. Therefore, we analysed whether there is an association between the total number of escape and compensatory mutations in Gag that we identified and the replication capacity of biological HIV-1 isolates obtained during the course of infection from our HLA-B*57/58:01 progressors and LTNPs (previously described in [25]). We observed a correlation between the number of mutations and an increasing replication capacity (R = 0.33, p = 0.04, Figure 2). This supports the idea that compensation of the fitness cost associated with HLA-B*57/58:01 CTL escape is the result of the accumulation of multiple mutations that increase viral fitness.

## Discussion

HLA-B*57 and HLA-B*58:01 are overrepresented in HIV-1 long-term nonprogressors, however, large differences in clinical disease course are observed among HLA-B*57/58:01 patients. Previously, we have shown that HLA-B*57/58:01 LTNPs and progressors have similar frequencies of HIV-1 Gag specific CTL responses and similar dynamics in escape mutations in HLA-B*57/58:01-restricted CTL epitopes [25]. Although escape mutations allow for evasion of CTL responses, the prevalence of escape mutations does not explain the differential disease course observed in these patients. CTL escape mutations in the HLA-B*57/58:01-restricted epitopes situated in Gag often impair viral replication and this fitness loss can be partially compensated for by additional mutations within or flanking these CTL epitopes [22]–[28]. We here studied whether the variation in clinical course of HIV-1 infection of HLA-B*57/58:01-positive individuals may be explained by differences in the number of mutations that restore fitness of viral variants that escaped from HLA-B*57/58:01-restricted CTL pressure.

The SH algorithm was used to study differences in Gag viral sequences obtained from HLA-B*57/58:01 negative and positive individuals, and from HLA-B*57/58:01 progressors and LTNPs. An important advantage of this approach is that it is unbiased toward certain amino acid positions that have previously been reported to be associated with HLA-B*57/58:01 or with a progressive disease course. Furthermore, the SH algorithm also allowed detection of very small differences in the frequency of amino acids between the two groups of sequences, whereas other specificity site detection methods would discard these due to lack of within-group conservation. Particularly in the case of the highly conserved Gag region the detection of very small differences in amino acid composition is crucial.

We observed that virus isolates from HLA-B*57/58:01 individuals, irrespective of their disease course, contained CTL escape mutations in the TW10 epitope (T242N and G248A) and the IW9 epitope (I147L), and a mutation adjacent to the KF11 epitope (S173T). Additionally, two other mutations (V159I and T280V) were observed specifically in viral variants from HLA-B*57/58:01 positive individuals; however, these mutations could not be related to escape from CTL pressure restricted by HLA-B*57/58:01. When comparing viral Gag sequences obtained late in infection from HLA-B*57/58:01 progressors and HLA-B*57/58:01 LTNPs, frequent changes in the amino acid sequence were observed for progressors at 5 positions: S126N, L215T, H219Q, M228I, N252H. An increase in replication kinetics was observed after introduction of two single mutations (L215T and H219Q) in the NL4-3.Ba-L virus containing the HLA-B*57/58:01-specific mutations. Nonetheless, the combination of all mutations associated with HLA-B*57/58:01 disease progression resulted in an even higher increase in replication kinetics. Introduction of a combination of mutations L215T and H219Q did not result in a significant increase in replication kinetics compared to the NL4-3.Ba-L virus containing the HLA-B*57/58:01-specific mutations, indicating that the combination of these two mutations does not account for the increase in replication kinetics observed for the virus containing the HLA-B*57/58:01-specific mutations. These findings suggest that multiple mutations are required to restore viral fitness after escape from HLA-B*57/58:01-restricted CTL responses [32]. To our knowledge, the S126N and L215T mutations have not been associated with an increase in viral fitness in HLA-B*57/58:01 CTL escape variants before, and we are the first to show that these mutations serve as compensatory mutations.

A previous study by Brockman *et al.* reported a higher frequency of mutations H219Q, I223V, M228I, G248A and N252H in combination with the HLA-B*57 T242N CTL escape mutation in sequences obtained from HLA-B*57 progressors than seen in sequences from HLA-B*57 non-progressors [27]. We observed that these mutations were able to increase the replication of NL4-3.Ba-L virus carrying the HLA-B*57/58:01-specific mutations; however, the replication capacity was still impaired as compared to the virus carrying all of the mutations associated with HLA-B*57/58:01 disease progression identified in our present study.

Our results and previous observations by others [32] suggest that compensatory mutations restore viral fitness in a cumulative manner. In sequences obtained from LTNPs late in the course of infection 1 or 2 compensatory mutations were present, whereas a higher number of mutations was usually observed in sequences obtained from progressors. The presence of a low number of compensatory mutations likely results in only a partial restoration of viral fitness. In line with this, our previous results show that biological viral variants from LTNPs obtained early and late in infection do not differ in their replication kinetics, whereas in HLA-B*57/58:01 progressors viral fitness increased over the course of infection [25]. Moreover, we here show a positive correlation between the number of HLA-B*57/58:01 escape and compensatory mutations and the replication capacity of HIV-1 biological viral variants obtained from HLA-B*57/58:01 patients, again suggesting there is a cumulative effect of compensatory mutations on viral replication fitness. Introduction of single mutations H219Q and L215T showed a significant increase in replication capacity, which may suggest that these mutations play a more prominent role in restoration of viral fitness.

The Gag protein is highly conserved and amino acid variation observed in Gag seems to be limited to specific positions, whereas other positions remain unchanged due to structural constraints. As a result, a high degree of conformity was observed in the amino acid positions described here and by previous studies [22], . This suggests that there is only a limited amount of positions within Gag that are capable of changing without a substantial fitness cost. Variation at these sites may serve as a general “coping” mechanism, and allow adaptation to changes in the capsid structure and capsid stability. Most of the compensatory mutations are located in one of three loops on the outer surface of capsid and are not involved in the formation of p24 hexamers and pentamers that make up the capsid lattice structure. The outer surface loops interact with host cellular proteins, like cyclophilin A (CypA) and restriction factor TRIM5α. The compensatory mutations, in particular H219Q, influence viral sensitivity to TRIM5α and reduce binding of capsid to CypA. This suggests that the introduction of compensatory mutations may influence HIV-1 replication through mechanisms affecting host factor dependency and sensitivity to intrinsic immunity [33]–[36].

In conclusion, we here identified 5 positions in the Gag region of HIV-1 that are more frequently mutated in sequences from HLA-B*57/58:01 progressors. The combination of these mutations can restore the replication fitness of CTL escape HIV-1 variants, which may explain the differences in disease progression. Furthermore, we observed that the total number of escape and compensatory mutations correlated with the replication fitness of biological HIV-1 isolates obtained from HLA-B*57/58:01 patients, suggesting that the replication fitness of HLA-B*57/58:01 escape variants is restored by accumulation of compensatory mutations.

## Materials and Methods

### Patients

Previously, we reported on 22 participants from the Amsterdam Cohort Studies (ACS) on HIV infection carrying the protective HLA-B*57 or HLA-B*58:01 allele who showed large differences in clinical disease course [25]. Ten patients who were followed longitudinally and from whom clonal HIV-1 viral sequences from at least two time points during follow-up were available were selected for the present study (Table 1). Seven individuals were HIV-1 infected (seroprevalent) at the moment of entry and three individuals seroconverted for HIV antibodies during active follow-up. All cohort participants had routine 3 monthly visits for blood donation and physical examination. Individuals who developed AIDS or who started with antiviral therapy within nine years after seroconversion or seroprevalent entry in the Amsterdam Cohort Studies were called progressors (n = 5; ACH19567 [P9], ACH18932 [P10], ACH18887 [P11], ACH13879 [P12], ACH11679 [P13]). Long-term non-progressors (LTNP) were those participants who had stable CD4+ T cell counts that were still above 400 cells/µl blood in the 10th year after SC or seroprevalent entry in the Amsterdam Cohort Studies or who had a CD4+ T cell decline less than 40*10^9^cells/l per year over a period of at least 10 years (n = 5; ACH19933 [L5], ACH19922 [L6], ACH19789 [L7], ACH19784 [L8], ACH19291 [L9]).

Two participants (L6 and L7) were heterozygous for the 32 base pair deletion in the CCR5 gene, whereas all other participants had a CCR5 wild type genotype. Typing of the HLA A and B alleles of all participants (Table 1) did not reveal other HLA types that were associated with a difference in disease progression.

### Ethics statement

The ACS has been conducted in accordance with the ethical principles set out in the declaration of Helsinki and written informed consent is obtained prior to data collection. This study was approved by the Amsterdam Medical Center institutional medical ethics committee.

### Viruses

Five HLA-B*57/58:01 progressors and five HLA-B*57/58:01 LTNPs were followed longitudinally. Clonal HIV-1 variants were isolated from patient PBMCs that were obtained early after seroconversion or study entry, at a time point as late as possible in the course of infection before the start of therapy and from PBMCs isolated at an in between time point (Table 1) [25]. From some patients virus isolation from PBMCs that were obtained early in the course of infection failed due to low viral loads at these time points. Clonal viral variants were obtained from single productively infected cells by cocultivation of PHA-stimulated PBMCs from healthy donors and serial dilutions with patient PBMC as described previously [37]. In brief, increasing numbers of patient PBMC were cocultivated with PHA-stimulated healthy donor PBMC in 96-well microtiter plates with four parallel microcultures per patient cell number. Every week, culture supernatants were tested for the presence of p24 antigen by an in-house p24 ELISA [38]. At the same time, one-third of the culture volume was transferred to a new 96-well plate and fresh PHA-stimulated PBMC from a healthy donor were added to propagate the culture. From the wells positive in the p24 antigen ELISA, virus stocks were grown and cryopreserved.

### DNA isolation, PCR amplification and sequencing

Viral DNA was isolated from cryopreserved PBMC that were infected with one clonal HIV-1 variant, using the L6 isolation method [39]. Gag DNA (positions 1−364) was amplified by a nested polymerase chain reaction (PCR) with outer primers Gag forward (5′-CGACGCAGGACTCGGCTTGCTG-3′; HXB2 nucleotides 684−705) and Gag outer reversed (5′-GCCTGTCTCTCAGTAC-3′; HXB2 nucleotides 2065−2080) and 2 different inner PCR primer combinations: Gag BssHII fw (5′-TGCTGAAGCGCCCGCACGGC-3′; HXB2 nucleotides 701−720) or Gag ClaI fw (5′-GGGAGAATTAGATCGATGGG-3′; HXB2 nucleotides 818−837) in combination with Gag p17 rev (5′-CAAAACTCTTGCCTTATGG-3′; HXB2 nucleotides 1859−1880) and Gag p17 fw (5′-TGCTAAACACAGTGGGGGGACAT-3′; HXB2 nucleotides 1349−1371) in combination with Gag ApaI rev (5′-TTCCTAGGGGCCCTGCAA-3′; HXB2 nucleotides 2000−2017).

PCR products were purified and sequenced with the ABI prism BigDye Terminator sequencing kit (Perkin Elmer, Froster City, California, USA) on an ABI 3130 XL DNA sequencer according to the manufacturer's protocol using the same PCR primers that were used for the nested PCR. DNA sequences were analyzed with Seqman software (DNAStar software package; Lasergene, Madison, Wisconsin, USA). The nucleotide sequences of the *gag* region were translated and edited with the BioEdit program (BioEdit v 7.0.5, Tom Hall, Ibis Therapeutics, Carlsbad, California, USA).

### Comparison of viral sequences with Sequence Harmony

To evaluate amino acid differences in Gag between patients positive and negative for HLA-B*57/58:01 and between HLA-B*57/58:01 positive LTNPs and HLA-B*57/58:01 progressors, the *gag* sequences obtained from these patients were analyzed with Sequence Harmony (SH) [29]–[31]. The SH algorithm is an entropy-based method, that detects positions with compositional differences within a multiple sequence protein alignment, as previously described [30] and in the online documentation on the web server (www.ibi.vu.nl/programs/seqharmwww). SH measures the overlap in distribution of amino acid types between two subgroups (*A* and *B*), in this case *gag* sequences obtained from HLA-B*57/58:01 and non- HLA-B*57/58:01 patients or HLA-B*57/58:01 LTNPs and HLA-B*57/58:01 progressors, at a certain position (*i*) in the sequence alignment as follows:
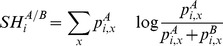
where 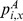 indicates the observed frequency in group A for amino acid type *x* at position *i* in the sequence and 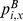 analogously for amino acid frequencies observed in group B sequences. The final SH score is calculated by 
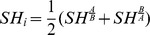
. Therefore, an SH score of 0 indicates amino acid positions that are specific for one of the sequence groups, whereas an SH score of 1 indicates an identical amino acid composition (i.e. complete overlap) at this alignment position between the two groups. To account for the unbalanced numbers of sequences available per patient (due to differing numbers of sample time points and numbers of sequences retreived per sample), we have also performed the SH analysis where we weighted each sequences by 1/N*_p_*, where N*_p_* is the number of sequences for patient *p* in the calculation of the SH score, such that each patient in total has equal weight in the SH analysis.

In the SH analyses performed here, a cut-off value of 0.90 was used to determine amino acid positions that are different between two groups of sequences. As the SH values are in a logarithmic scale, one could interpret the 0.90 cut-off as an approximate 10% difference in amino acid distribution. The high cut-off allows for detection of relatively small differences against a background of overall high conservation. Note that conservation overall or within a group of sequences is not explicitly taken into account in the SH analysis. The cut-off of 0.9 for the SH score implies that sites are already of interest when the frequency of occurrence of some amino acid types at a certain position changes. Positions where the difference between the two groups of sequences was located at a minor amino acid for both groups, or where the aberrant amino acid was identical to our NL4-3.Ba-L backbone were excluded from the analysis.

Z-scores were calculated based on random SH scores from 100-fold random shuffling of sequence labels (i.e. random re-distribution of sequences over two groups of the same size as the patient groups in the analysis); we assumed a normal distributions of these random SH scores based on previous tests [31]. P values were estimated based on the Z-scores and correction for multiple testing was applied based on the number of columns in the multiple sequence alignment, which was around 450. In this calculation, Z<−6 (absolute value) corresponds to an uncorrected p value of <1e-9, therefore a Z<−10 (absolute value) corresponds to a *corrected* p-value of <1e-9. In the discussion of the results, we will for simplicity state p<0.0001 for Z<−10 (absolute value).

### Cells

293T cells (ATCC) were cultured in Dulbecco Modified Eagle's Medium (DMEM) supplemented with 10% inactivated fetal calf serum (FCS; Hyclone, Logan, Utah, USA), 100 U/ml penicillin (Gibco, Paisly, Scotland, UK) and 100 µg/ml streptomycin (Gibco) in a humidified 10% CO_2_ incubator at 37°C.

PBMC from 10 healthy donors were isolated from buffy coats by Ficoll density centrifugation, pooled and cryopreserved. After thawing, the PBMC pool was stimulated for three days in Iscoves' Modified Dulbecco's Medium (IMDM) supplemented with 10% FCS, 100 U/ml penicillin, 100 µg/ml streptomycin, 5 µg/ml ciproxin (Bayer, Mijdrecht, the Netherlands) and 1 µg/ml phytohaemagglutinin (PHA; Remel Europe, Dartford, England, UK) at a cell density of 5×10^6^ cells/ml in a humidified 10% CO_2_ incubator at 37°C. PBMC cultures were continued in IMDM supplemented with 10% FCS, 20 U/ml recombinant interleukin 2 (rIL2; Chiron Benelux, Amsterdam, the Netherlands, 5 µg/ml polybrene (Sigma, Zwijndrecht, the Netherlands), 100 U/ml penicillin and 100 µg/ml streptomycin at a cell density of 1×10^6^/ml in a humidified 10% CO_2_ incubator at 37°C.

### Construction of replication competent molecular viral clones

For the construction of HIV-1 variants that contain mutations in the Gag protein, the *gag* region of the molecular clone NL4-3.Ba-L was removed using restriction enzymes BssHII and ApaI and then cloned into the pGEM T easy vector (Promega, Madison, Wisconsin, USA). Mutations in the *gag* region were introduced using site directed mutagenesis as described by the manufacturer (Quick exchange kit, Stratagene). The *gag* insert was sequenced to confirm successful mutagenesis using primers T7 (5′-TAATACGACTCACTATAGGG-3′) and SP6 (5′-GATTTAGGTGACACTATAG-3′).

For the production of replicating NL4-3.Ba-L clones containing the desired mutations, the mutated *gag* insert was ligated into the full length molecular NL4-3.Ba-L clone using BssHII and ApaI restriction sites. The full-length NL4-3.Ba-L molecular clones were then transfected into 293T cells using the calcium phosphate method [40]. Virus production from the 293T cells was analyzed at day 3 and day 7 after transfection using an in house p24 ELISA [38]. Virus stocks were grown on PHA-stimulated PBMC and virus titers were determined by 50% tissue culture infectious dose (TCID50) as previously described [37]. The *gag* regions of obtained viruses were sequenced as described above to confirm the introduction of mutations.

### Viral replication assay

To determine the viral replication rates of the different HIV-1 molecular clones and biological variants, 2×10^6^ PHA-stimulated pooled PBMC were inoculated with 100 TCID50 per virus variant for 2 hours at 37°C in a shaking water bath in a total volume of 1.5 ml. Subsequently, the inoculated PBMC were washed with 5 ml of IMDM supplemented with 10% FCS, and cultured in IMDM supplemented with 10% FCS, 20 U/ml rIL2, 5 µg/ml polybrene, 100 U/ml penicillin and 100 µg/ml streptomycin at a cell density of 1×10^6^ per ml in a humidified 10% CO_2_ incubator at 37°C. On day 5, 8, 11 and 14 after inoculation, 1×10^6^ fresh PHA-stimulated pooled PBMC in 1 ml of IMDM culture medium supplemented with 10% FCS, 20 U/ml rIL2, 5 µg/ml polybrene, 100 U/ml penicillin and 100 µg/ml streptomycin were added to the cultures. Samples for determination of p24 antigen production (75 µl) were harvested every day after inoculation. All samples were tested for p24 antigen production simultaneously at the end of the experiment using an in-house p24 ELISA [38].

On the last day of the replication experiments, DNA from every well was isolated using the L6 method [39] and the *gag* region of all samples was sequenced as described above to confirm the presence of mutations. The replication rates of all viruses were tested at least in twofold and in 2 independent experiments for which the same pool of healthy donor PBMC was used. P24 values were normalized to the mean p24 values observed for the constructed NL4-3.Ba-L virus carrying mutations associated with the presence of HLA-B*57/58:01. The area under the curve for the replication curves spanning day 2−17 was calculated and normalized for the constructed NL4-3.Ba-L virus carrying mutations associated with the presence of HLA-B*57/58:01. Statistical significance of differences in the p24 production on day 11 and the area under the curve were tested with the unpaired Student's T test. In Figure 1 and Figure S1 data from 1 representative experiment are shown.

To test the correlation between the number of escape and compensatory mutations in Gag with the replication fitness of biological HIV-1 variants obtained from HLA-B*57/58:01 progressors and LTNPs, a Spearman correlation test was performed. P values <0.05 were considered significant. Statistical analyses were preformed using Graphpad Prism version 5 and SPSS version 19.

## Supporting Information

Figure S1 **Sequence variation in Gag affects viral replication fitness.** A. Replication kinetics of constructed NL4-3.Ba-L viral variants containing mutations associated with HLA-B*57/58:01 in the absence or presence of compensatory mutations. The area under the curve (day 2–17) was calculated and normalized mean AUCs were compared using the unpaired Student's T test. Statistical significance compared to the mutant virus carrying the mutations associated with the presence of HLA-B*57/58:01 are denoted in red, and significance compared to the virus carrying all mutations associated with the presence of HLA-B*57/58:01 and with disease progression is shown in blue. Statistical significance is indicated as follows: * p<0.05, ** p<0.01, *** p<0.0001. Error bars represent 2.5–97.5 percentiles. Data from one representative experiment are shown. B. Replication kinetics of constructed NL4-3.Ba-L viral variants containing mutations associated with HLA-B*57/58:01 in the absence or presence of compensatory mutations described by Brockman *et al*. The area under the curve (day 2–17) was calculated and normalized mean AUCs were compared using the unpaired Student's T test. Statistical significance compared to the mutant virus carrying the mutations associated with the presence of HLA-B*57/58:01 are denoted in red, and significance compared to the virus carrying all mutations associated with the presence of HLA-B*57/58:01 and with disease progression is shown in blue. Statistical significance is indicated as follows: * p<0.05, ** p<0.01, *** p<0.0001.Error bars represent 2.5 – 97.5 percentiles. Data from one representative experiment are shown.
(TIF)

Table S1 **Amino acid sequence variation in Gag.** A. Sequence variation within Gag located at amino acid positions associated with the presence of HLA-B*57/5801 or disease progression as identified with SH in sequences obtained from LTNPs from various time points during the course of HIV-1 infection. B. Sequence variation within Gag at amino acid positions associated with the presence of HLA- B*57/5801 or disease progression as identified with SH in sequences obtained from progressors from various time points during the course of HIV-1 infection.
(DOC)
