# Supplementary figures and images for: HIV-1 Replication Fitness of HLA-B*57/58:01 CTL Escape Variants Is Restored by the Accumulation of Compensatory Mutations in Gag
Source: PLoS One. 2013 Dec 5;8(12):e81235. doi: 10.1371/journal.pone.0081235 (PMC3855271; doi:10.1371/journal.pone.0081235)

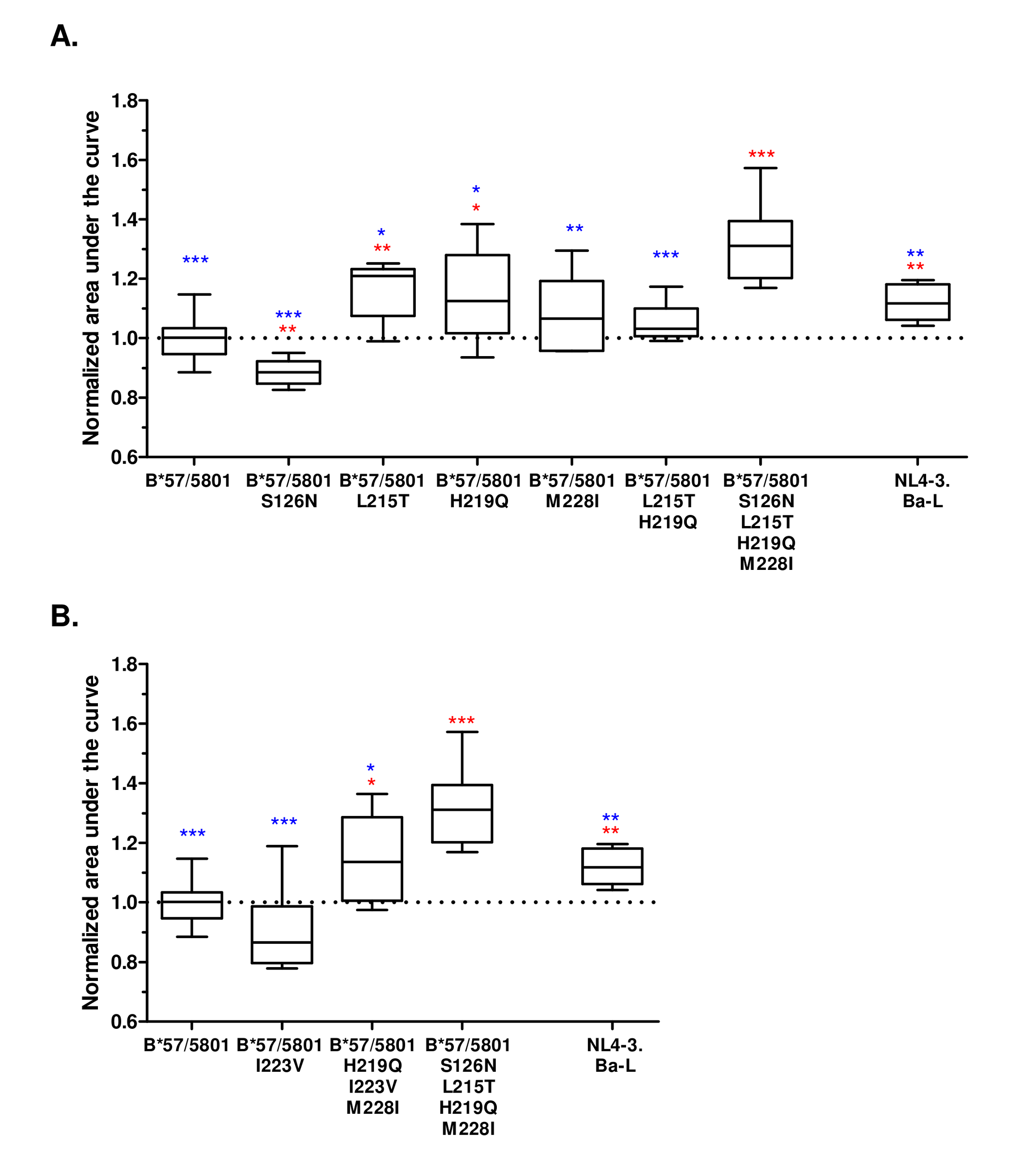

Supplement: Figure S1 — Sequence variation in Gag affects viral replication fitness. A. Replication kinetics of constructed NL4-3.Ba-L viral variants containing mutations associated with HLA-B*57/58:01 in the absence or presence of compensatory mutations. The area under the curve (day 2–17) was calculated and normalized mean AUCs were compared using the unpaired Student's T test. Statistical significance compared to the mutant virus carrying the mutations associated with the presence of HLA-B*57/58:01 are denoted in red, and significance compared to the virus carrying all mutations associated with the presence of HLA-B*57/58:01 and with disease progression is shown in blue. Statistical significance is indicated as follows: * p<0.05, ** p<0.01, *** p<0.0001. Error bars represent 2.5–97.5 percentiles. Data from one representative experiment are shown. B. Replication kinetics of constructed NL4-3.Ba-L viral variants containing mutations associated with HLA-B*57/58:01 in the absence or presence of compensatory mutations described by Brockman et al. The area under the curve (day 2–17) was calculated and normalized mean AUCs were compared using the unpaired Student's T test. Statistical significance compared to the mutant virus carrying the mutations associated with the presence of HLA-B*57/58:01 are denoted in red, and significance compared to the virus carrying all mutations associated with the presence of HLA-B*57/58:01 and with disease progression is shown in blue. Statistical significance is indicated as follows: * p<0.05, ** p<0.01, *** p<0.0001.Error bars represent 2.5 – 97.5 percentiles. Data from one representative experiment are shown. (TIF) [file pone.0081235.s001.tif]
