# Supplementary material for: HIV-1 Replication Fitness of HLA-B*57/58:01 CTL Escape Variants Is Restored by the Accumulation of Compensatory Mutations in Gag
Source: PLoS One. 2013 Dec 5;8(12):e81235. doi: 10.1371/journal.pone.0081235 (PMC3855271; doi:10.1371/journal.pone.0081235)
Supplement: Table S1 — Amino acid sequence variation in Gag. A. Sequence variation within Gag located at amino acid positions associated with the presence of HLA-B*57/5801 or disease progression as identified with SH in sequences obtained from LTNPs from various time points during the course of HIV-1 infection. B. Sequence variation within Gag at amino acid positions associated with the presence of HLA- B*57/5801 or disease progression as identified with SH in sequences obtained from progressors from various time points during the course of HIV-1 infection. (DOC) [file pone.0081235.s002.doc]

**Table S1A.** Sequence variation within Gag located at amino acid positions associated with the presence of HLA-B*57/5801 or disease progression as identified with SH in sequences obtained from LTNPs from various time points during the course of HIV-1 infection.

| **Patient** | **Time after SC or study entry (months)** | **number of sequences** | **S126** | **I147** | **V159** | **S173** | **L215** | **H219** | **M228** | **T242** | **G248** | **N252** | **T280** |
| --- | --- | --- | --- | --- | --- | --- | --- | --- | --- | --- | --- | --- | --- |
| L5 | 89 | 4/6 | N | L | . | T | . | . | . | N | . | . | . |
|  |  | 2/6 | - | L | . | T | . | . | . | N | . | . | . |
|  | 177 | 6/6 | N | L | . | T | . | . | . | N | . | . | . |
| L6 | 17 | 2/5 | . | . | I | . | M | . | . | N | . | S | . |
|  |  | 1/5 | . | . | I | . | M | . | . | N | . | S | I |
|  |  | 1/5 | . | . | . | . | M | . | . | N | . | S | . |
|  |  | 1/5 | . | . | . | . | M | . | I | . | A | S | . |
|  | 114 | 6/15 | . | L | I | . | M | . | . | N | . | S | . |
|  |  | 2/15 | . | L | I | . | M | . | . | N | . | . | . |
|  |  | 2/15 | . | . | I | . | M | . | I | N | T | S | . |
|  |  | 2/15 | . | . | I | . | M | . | I | N | T | . | . |
|  |  | 2/15 | . | . | I | . | M | . | I | . | A | . | . |
|  |  | 1/15 | . | . | I | . | M | . | . | N | . | . | . |
| L7 | 26 | 2/5 | . | L | . | . | . | Q | . | N | A | . | V |
|  |  | 1/5 | . | L | I | . | . | Q | . | N | A | . | V |
|  |  | 1/5 | . | L | . | . | . | Q | . | N | A | . | I |
|  |  | 1/5 | . | L | . | . | . | . | . | N | A | . | V |
|  | 78 | 5/6 | . | L | I | . | . | Q | . | N | A | . | V |
|  |  | 1/6 | . | L | I | T | . | . | . | N | A | S | V |
|  | 102 | 6/8 | . | L | I | T | . | . | . | N | A | S | V |
|  |  | 1/8 | . | L | I | . | . | . | . | N | A | S | V |
|  |  | 1/8 | **.** | . | . | . | . | . | . | N | A | S | V |
|  | 136 | 12/12 | . | L | I | T | . | . | . | N | A | S | V |
| L8 | 70 | 1/2 | . | L | . | . | M | Q | . | N | . | S | I |
|  |  | 1/2 | . | L | . | . | M | Q | . | N | . | S | - |
|  | 91 | 7/9 | . | L | . | . | . | . | . | N | A | . | I |
|  |  | 1/9 | . | L | . | . | . | . | . | S | A | . | I |
|  |  | 1/9 | . | L | . | T | . | . | . | N | . | . | . |
|  | 137 | 3/6 | . | L | . | . | . | . | . | N | A | . | V |
|  |  | 1/6 | . | L | . | . | . | . | . | N | A | . | - |
|  |  | 2/6 | . | L | I | T | . | . | . | N | . | . | V |
| L9 | 42 | 1/1 | N | . | . | . | . | . | . | N | . | . | . |
|  | 59 | 1/1 | N | . | . | . | . | . | . | N | . | . | . |
|  | 77 | 6/6 | . | . | . | . | . | . | . | N | . | . | . |

**Table S1B.** Sequence variation within Gag at amino acid positions associated with the presence of HLA-

| **Patient** | **Time after SC or study entry (months)** | **number of sequences** | **S126** | **I147** | **V159** | **S173** | **L215** | **H219** | **M228** | **T242** | **G248** | **N252** | **T280** |
| --- | --- | --- | --- | --- | --- | --- | --- | --- | --- | --- | --- | --- | --- |
| P9 | 9 | 5/8 | . | M | . | . | V | . | . | N | A | H | . |
|  |  | 3/8 | . | M | . | . | V | . | L | N | A | H | . |
|  | 78 | 2/3 | . | L | . | T | . | . | . | N | A | H | . |
|  |  | 1/3 | . | L | . | T | . | . | . | N | A | H | A |
| P10 | 69 | 4/7 | N | L | I | T | T | Q | . | N | . | S | V |
|  |  | 2/7 | . | L | I | T | T | Q | . | N | . | S | V |
|  |  | 1/7 | - | - | - | T | T | Q | . | N | . | S | V |
|  | 84 | 5/7 | N | L | I | T | T | Q | I | N | T | S | V |
|  |  | 2/7 | N | L | I | T | T | Q | . | N | . | S | V |
|  | 123 | 7/9 | N | L | I | T | T | Q | I | N | T | S | V |
|  |  | 1/9 | - | - | I | T | T | Q | . | N | . | S | V |
|  |  | 1/9 | - | - | - | - | T | Q | . | N | . | S | V |
| P11 | 3 | 2/2 | . | . | . | . | . | . | . | N | . | H | . |
|  | 25 | 7/9 | . | L | . | . | . | . | . | N | . | H | . |
|  |  | 1/9 | . | . | . | . | . | . | . | N | . | H | . |
|  |  | 1/9 | . | M | I | . | . | . | . | N | . | H | . |
|  | 32 | 15/23 | . | L | . | . | . | . | . | N | . | H | . |
|  |  | 1/23 | . | L | . | . | . | . | . | N | A | H | . |
|  |  | 3/23 | . | M | . | . | . | . | . | N | A | H | . |
|  |  | 2/23 | . | M | I | . | . | . | . | N | A | H | . |
|  |  | 1/23 | N | M | I | . | . | . | . | N | A | H | . |
|  |  | 1/23 | . | . | . | . | . | . | . | N | . | H | . |
|  | 69 | 6/13 | . | L | I | . | . | . | I | N | A | H | . |
|  |  | 4/13 | . | L | . | T | . | . | . | N | A | H | . |
|  |  | 1/13 | . | M | . | . | . | . | . | N | A | H | . |
|  |  | 1/13 | . | L | I | . | . | . | I | N | A | . | . |
|  |  | 1/13 | . | L | . | . | . | . | I | N | A | . | . |
| P12 | 0.4 | 1/5 | N | L | I | T | I | . | L | N | . | S | . |
|  |  | 1/5 | K | L | I | T | I | . | L | N | . | S | . |
|  |  | 1/5 | K | L | I | T | M | . | L | N | . | S | . |
|  |  | 1/5 | K | L | I | T | I | . | L | N | . | S | V |
|  |  | 1/5 | K | L | I | T | M | . | L | N | . | S | V |
|  | 48 | 4/11 | K | L | I | T | T | . | L | N | . | G | V |
|  |  | 1/11 | N | L | I | T | T | . | L | N | . | G | V |
|  |  | 5/11 | K | L | I | T | T | . | L | N | . | S | V |
|  |  | 1/11 | N | L | I | T | T | . | L | N | . | S | V |
|  | 74 | 9/12 | K | L | I | T | T | Q | L | N | . | S | V |
|  |  | 1/12 | K | L | I | T | I | Q | L | N | . | S | V |
|  |  | 2/12 | K | L | I | T | T | . | L | N | . | S | V |
| P13 | 3 | 5/6 | . | M | I | . | . | . | I | N | A | S | . |
|  |  | 1/6 | . | L | . | . | . | . | I | N | A | S | . |
|  | 46 | 10/10 | . | M | I | . | . | . | I | N | A | S | . |
|  | 88 | 3/6 | . | M | I | . | . | Q | . | N | A | H | S |
|  |  | 2/6 | . | M | I | . | . | Q | L | N | A | H | S |
|  |  | 1/6 | . | M | I | . | . | Q | I | N | A | H | S |

B*57/5801 or disease progression as identified with SH in sequences obtained from progressors from various time points during the course of HIV-1 infection.
